# Four-dimensional ultrasound guided embryo transfers improve live birth rates when compared to the clinical touch technique: a randomised controlled trial

**DOI:** 10.1038/s41598-023-41313-z

**Published:** 2023-09-08

**Authors:** L. Nancarrow, Nicola Tempest, S. Lane, R. Homburg, R. Russell, D. K. Hapangama

**Affiliations:** 1 Department of Women’s and Children’s Health, Institute of Life Course and Medical Sciences, Centre for Women’s Health Research, Member of Liverpool Health Partners, University of Liverpool, Liverpool, L8 7SS UK; 2 Hewitt Centre for Reproductive Medicine, Liverpool Women’s NHS Foundation Trust, Liverpool, L8 7SS UK; 3 Liverpool Women’s NHS Foundation Trust, Member of Liverpool Health Partners, Liverpool, L8 7SS UK; 4 Department of Biostatistics, Institute of Life Course and Medical Sciences, Member of Liverpool Health Partners, University of Liverpool, Liverpool, UK

**Keywords:** Radiography, Health care, Medical research

## Abstract

Most aspects of in-vitro fertilisation (IVF) have changed dramatically since introduction, but embryo transfer (ET) technique remains largely unaltered. We aimed to determine whether four-dimensional ultrasound guided embryo transfers (4D UGET) could improve pregnancy rates when compared with clinical touch technique (CTT). This was a single centre open labelled randomised controlled trial in a tertiary fertility centre in the UK. 320 women were randomised on the day of single ET. The primary outcome was clinical pregnancy rate (CPR), secondary outcomes included live birth rate (LBR), biochemical pregnancy rate (BPR), miscarriage, pregnancy of unknown location (PUL) and ectopic pregnancy. 4D-UGET resulted in significantly higher CPR [50% vs 36% p = 0.02, OR 1.78 (1.12–2.84)] and LBR [41% vs 28%, p = 0.02, OR 1.77 (1.09–2.87)] when compared to CTT technique. Miscarriage (p = 0.49), PUL (p = 0.14) and ectopic pregnancy (p = 0.96) were similar between the two groups. LBR, from this trial, are significantly higher than the current UK average (41% vs 24%). 4D UGET allows for superior imaging of the uterine cavity, whilst tailoring the embryo deposition point specifically to the patient. Further RCTs are required to determine if these results can be replicated in other units and whether 4D UGET is superior to 2D UGET.

## Introduction

Most aspects of in-vitro fertilisation (IVF) have developed and changed dramatically since their introduction^1^. In contrast, the technique of embryo transfer (ET) remains largely unaltered^2,3^. The current clinical pregnancy rate (CPR) per ET is 28.5%^4^, denotating that over two thirds of ETs are unsuccessful in achieving a pregnancy. This may be due to imperfect embryos (e.g. chromosomal abnormalities^5^), defective endometrium (e.g. unreceptive and deficient endometrium^5^), or potentially, a suboptimal ET technique^5,6^.

The clinical touch technique (CTT), due to the blind nature of the procedure, can lead to inadvertent trauma with the catheter disrupting the endometrium^7,8^. This may induce high frequency uterine contractions, resulting in displacement of the embryo^9^ and a negative impact on CPR and live birth-rate (LBR)^10,11^.

In 2016, a Cochrane review (n = 5859) demonstrated that transabdominal (TA) two-dimensional (2D) ultrasound guided embryo transfers (UGET) led to an increased LBR compared with the CTT (OR 1.47, 95% CI 1.30 to 1.65; 13 trials; n = 5859 women; I^2^ = 74%; low‐quality evidence)^12^. UGET is thus recommended by the National Institute of Clinical Excellence (NICE) in the United Kingdom (UK) and is used in 77% of ETs worldwide^13^. A significant proportion of units still employ the CTT, likely due to the low quality data, with high heterogeneity, that was used to draw conclusions from in the 2016 Cochrane review^12^. Our unit provided data from the largest trial included in the 2016 review, and our local data did not demonstrate a significant difference between CTT and UGET in LBR^14^.

TA three-dimensional (3D) ultrasound has been shown to confirm correct placement of a trial catheter prior to ET in a feasibility study, but authors did not subsequently perform the ETs under ultrasound guidance^15^. A further observational study also reported the ability of 3D and 4D ultrasound to ensure correct catheter placement via the TA route with a resulting increase in pregnancy rate of 10% with 3D/4D UGETs and deposition of the embryo at maximal implantation potential (MIP) point^16^, when compared with 2D scans^17^.

**Figure 1:**
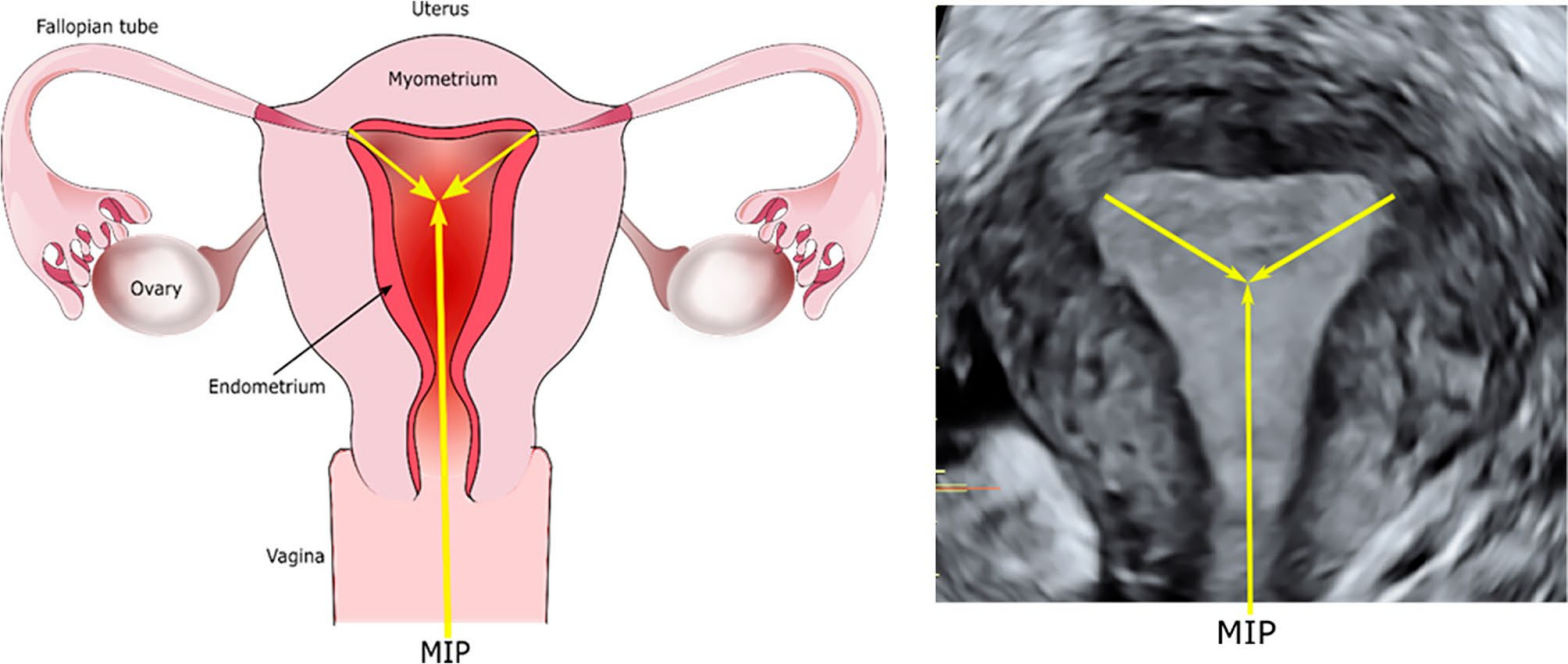
Maximal implantation potential point.

4D ultrasound allows for a real-time 3D view of the uterine cavity, which negates the delays that repetitive 3D sweeps would require, whilst still obtaining the improved spatial awareness of 3D imaging of the uterine cavity^18^. This provides an accurate view of the MIP, allowing embryo deposition at this point and potentially leading to improved ET outcomes^16^ (Fig. 1).

The primary aim of this project was to determine if 4D UGETs result in higher CPR, when compared with the CTT.

## Results

**Figure 2:**
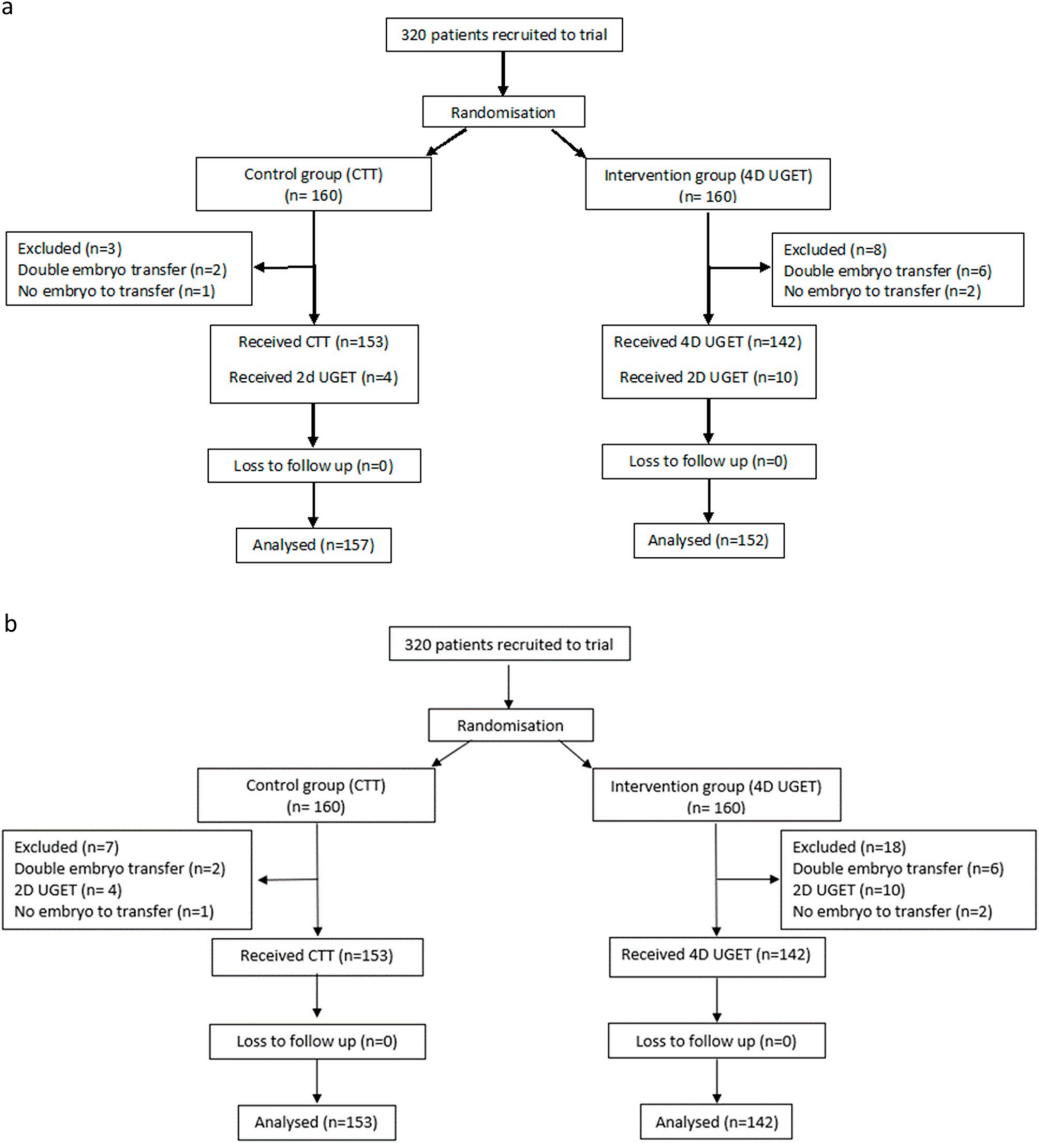
Flow diagram of recruitment and exclusions, (**a**) intention to treat, (**b**) per protocol.

320 patients were recruited over 17 months and randomised to the intervention (n = 160) and control (n = 160) groups (Fig. 2). The demographic and baseline cycle characteristics were comparable between the two groups (Table 1). All of the 4D UGET were performed by one practitioner and the majority (95%) of the CTT were also performed by the same practitioner.

**Table 1:** Baseline characteristics, intention to treat (ITT) and per protocol analysis CTT versus 4D UGET.

	ITT	ITT	P-value	Per protocol	Per protocol	P-value
	CTT (n = 157)	4D UGET (n = 152)		CTT (n = 153)	4D UGET (n = 142)	
Age (years) [mean ± SD]	33.96 [4.17]	33.24 [3.88]	0.12	34.04 [4.14]	33.12 [3.86]	0.05
BMI (kg/m^2^) [mean ± SD]	25.48 [5.39]	24.95 [3.66]	0.32	25.43 [5.40]	24.85 [3.58]	0.29
Duration of infertility (years) [mean ± SD]	3.70 [2.28]	3.68 [2.02]	0.93	3.68 [2.29]	3.66 [2.02]	0.94
Type of infertility						
Primary [n, %]	77 (49)	75 (49)	0.96	75 (49)	73 (51)	0.68
Secondary [n, %]	90 (51)	77 (51)		78 (51)	69 (49)	
Causes of infertility [n, %]*						
Male factor	58 (37)	59 (39)	0.73	55 (36)	54 (38)	0.71
Unexplained	43 (27)	46 (30)	0.58	42 (27)	45 (32)	0.43
PCOS/ovulatory	28 (18)	24 (16)	0.63	28 (18)	22 (15)	0.52
Tubal disease	11 (7)	13 (9)	0.61	11 (7)	11 (8)	0.87
Endometriosis	8 (5)	6 (4)	NA	8 (5)	6 (4)	NA
Low AMH	6 (4)	5 (3)	NA	6 (4)	5 (4)	NA
Same sex	6 (4)	5 (3)	NA	6 (4)	4 (3)	NA
Age	4 (3)	3 (2)	NA	4 (3)	3 (2)	NA
Single	3 (2)	1 (1)	NA	3 (2)	1 (1)	NA
Surrogate	2 (1)	1 (1)	NA	2 (1)	1 (1)	NA
AMH [mean ± SD]	25.35 [23.15]	25.78 [23.85]	0.88	25.31 [23.35]	25.73 [24.37]	0.88
Number of previous IVF/ICSI attempts [mean ± SD]	1.31 [0.69]	1.36 [0.85]	0.57	1.31 [0.69]	1.32 [0.77]	0.84
Number of previous ETs [mean ± SD]	1.31 [1.76]	1.21 [1.53]	0.10	1.3 [1.76]	1.18 [1.48]	0.51
Type of stimulation cycle						
Agonist [n, %]	13 (8)	9(6)	0.53	12 (8)	8 (6)	0.59
Antagonist [n, %]	135 (86)	137(90)		132 (86)	128 (90)	
Embryo recipient [n, %]	9(6)	6(4)		9 (6)	6 (4)	
Oocytes retrieved [mean ± SD]	14.7 [8.62]	13.6 [7.57]	0.23	14.8 [8.69]	13.7 [7.75]	0.27
Type of ET						
Fresh [n, %]	92 (59)	101 (66)	0.15	63 (41)	48 (34)	0.19
Frozen [n, %]	65 (41)	51 (34)		90 (59)	94 (66)	
Embryo quality**						
Good [n, %]	95(61)	98(64.5)	0.77	93 (61)	90 (63)	0.89
Average [n, %]	43(27)	38(25)		42 (27)	37 (26)	
Poor [n, %]	19(12)	16(10.5)		18 (12)	15 (11)	

*ICSI* intracystoplasmic sperm injection.

*Percentages do not add up to 100 as some women had more than one pathology leading to infertility.

**See Supplementary Fig. 1 for embryo quality grading tool.

### Clinical outcomes

4D UGET led to a significantly higher CPR when compared with the CTT group (per protocol 50% vs 36%, p = 0.02, ITT 47% vs 36%, p = 0.04). The 4D UGET group also had a higher LBR and BPR compared with the CTT group (per protocol 41% vs 28%, p = 0.02, ITT 39% vs 28%, p = 0.04 and per protocol 59% vs 46%, p = 0.02, ITT 57% vs 45%, p = 0.04 respectively) (Table 2). Logistic regression, adjusting for age and BMI, continued to reveal statistical significance between control and 4D UGET across CPR, LBR and BPR. The time taken to perform 4D UGET was significantly longer than the CTT ET (Table 2). No differences were seen in the other clinical outcomes between the two groups.

**Table 2:** Clinical outcome measures, Intention to treat (ITT) and per protocol analysis CTT versus 4D UGET.

	ITT CTT (n = 157)	ITT 4D UGET (n = 152)	ITT P-value	ITT odds ratio (95% confidence interval)	Per protocol CTT (n = 153)	Per protocol 4D UGET (n = 142)	Per protocol P-value	Per protocol odds ratio (95% confidence interval)
CPR [n, %]	56 (36)	72 (47)	0.04	1.62 (1.03–2.56)	55 (36)	71 (50)	0.02	1.78 (1.12–2.84)
LBR [n, %]	44 (28)	59 (39)	0.04	1.63 (1.01–2.63)	43 (28)	58 (41)	0.02	1.77 (1.09–2.87)
BPR [n, %]	71 (45)	87 (57)	0.04	1.62 (1.03–2.54)	70 (46)	84 (59)	0.02	1.71 (1.08–2.72)
Miscarriage [n, %]	12 (8)	12 (8)	0.40	0.69 (0.29–1.65)	12 (8)	12 (8)	0.49	0.73 (0.30–1.79)
Ectopic pregnancy [n, %]	1 (0.6)	1 (0.7)	0.98	1.03 (0.06–16.6)	1 (0.6)	1 (0.7)	0.96	1.08 (0.07–17.3)
PUL [n, %]	0 (0)	2 (1.3)	0.15	1.01 (0.99–1.03)	0 (0)	2 (1.4)	0.14	1.01 (0.99–1.03)
TOP [n, %]	0 (0)	1 (0.7)	0.31	1.01 (0.99–1.02)	0 (0)	1 (0.7)	0.30	1.01 (0.99–1.02)
Duration of procedure (minutes) [mean ± SD]	10.45 [2.42]	16.19 [5.11]	< 0.01	NA	10.28 [2.18]	15.77 [2.62]	< 0.01	NA

### Patient satisfaction

No statistical difference was noted with regards to patient discomfort or satisfaction between the two procedures (Table 3). However, those in the 4D UGET arm of the trial were significantly more likely to write a comment (p < 0.001), and for the comment to be positive (p < 0.001), following the procedure. 87/142 (61%) women commented on their 4D UGET, 81 (93%) complimentary and 11/153 (7%) commented in the CTT group, 4 (36%) complimentary. In the comments, 36 women stated their preference of 4D UGET versus CTT ET.

**Table 3:** Patient satisfaction, Intention to treat (ITT) and per protocol analysis CTT versus 4D UGET.

	ITT CTT (n = 157)	ITT 4D UGET (n = 152)	ITT P-value	Per protocol CTT (n = 153)	Per protocol 4D UGET (n = 142)	Per protocol P-value
Patient comfort [mean ± SD]	4.41 (0.82)	4.25 (0.85)	0.11	4.44 (0.77)	4.31 (0.80)	0.17
Patient satisfaction [mean ± SD]	4.93 (0.38)	4.98 (0.14)	0.12	4.93 (0.39)	4.99 (0.12)	0.08

### Clinicians view

Ease of performing the procedure was not rated differently between the groups, however, 14 transfers were converted to 2D ultrasound guidance (3%) in the control group and 10 (7%) in the intervention group. Those that were converted to 2D ultrasound in the control group were due to difficultly experienced navigating the uterocervical angle during CTT. In the intervention group conversion was due to problematic visualisation of the uterine cavity using 4D ultrasound due to the uterus being axial in position or due to a thin endometrium. Conversion to 2D US allowed better visualisation of the catheter tip at the time of transfer in this situation.

## Discussion

### Main findings

4D UGET leads to a statistically significant increase in CPR, LBR and BPR when compared with the CTT. This is the first RCT examining the clinical efficacy of 4D UGET and the 14% increase in CPR we observed is in concordance with the previous observational study reporting a 10% increase in CPR when 4D UGET was implemented into routine practice^17^.

### Interpretation

Patients included in our 4D UGET group achieved a LBR of 41%, almost double that of the current national LBR per single ET of 24%^19^. Our recent survey on ET practice in the UK suggested that the majority (85%) of clinics in the UK are using 2D ultrasound guidance with only a small proportion still using the CTT^20^. Therefore, we postulate the observed increase in LBR of the 4D UGET group in our study to be as a result of optimal deposition of the embryo at the MIP in comparison to the blind, less ideal embryo deposition utilising the CTT.

**Figure 3:**
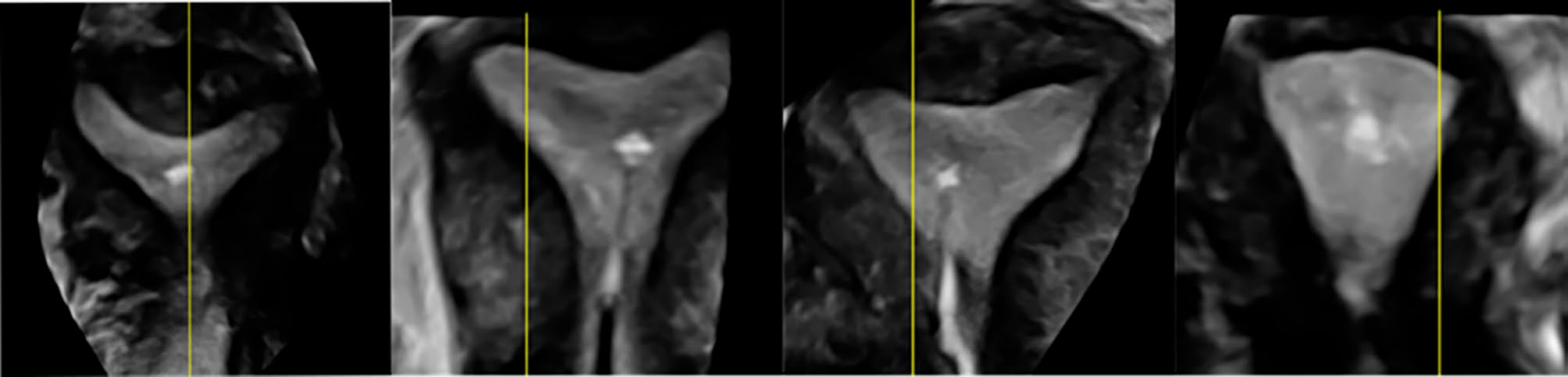
Variances in uterine cavity shape of 4 patients included in the trial highlighting the importance of a patient specific approach. Echobright areas show embryo deposition.

Previous studies using 2D ultrasound proposed the best embryo deposition point to lie within 0.5–2 cm from the fundus of the uterine cavity, which is further endorsed by recent reviews with recommended deposition in the upper/middle third of the uterine cavity (between 1 and 2 cm from the fundus)^21,22^. However, the length of the uterine cavity is dependent on the patient, phase of the menstrual cycle and stimulation protocol^23–25^, therefore, fixing a set distance from the fundus for embryo deposition is unlikely to demarcate the optimum location for all patients. Embryo deposition at the MIP under 4D guidance allows for a patient-specific personalised approach to support embryo implantation (Fig. 3).

Although there is a dearth of available evidence for 4D UGET, a number of previous studies have highlighted the benefit of obtaining 3D views of the endometrial cavity during ET^15,26,27^. The only published RCT comparing 3D and 2D UGET showed no difference in CPR between the 2 methods^28^, yet the study did not assess the duration of procedure and did not have sufficient power to assess the differences in the subsets of women involved. Procedure associated delay may be exacerbated by additional trauma from repeated catheter adjustment required with 3D UGET^29^. Therefore, our study compared CTT with 4D UGET, a technique which removes these deficiencies identified with 3D UGET.

The largest 4D UGET study to date, reported by Gergely and colleagues was a retrospective observational study, and the authors found an increase in pregnancy rate of 10% compared with 2D UGET when 3D/4D ultrasound guidance was implemented^17^. Unfortunately, the impact of transfer catheters, clinicians, embryo age and laboratory and ultrasound advances, during their five-year data collection period, were not considered^16,30^. This reduces the generalisability of the results from their study.

There are many postulated benefits of 4D UGET, which include improved patient experience and comfort. However, this was not reflected in the patient satisfaction/comfort scores, presumably due to a large proportion of the women not having experienced any other form of transfer. However, the large number of positive responses in the free text comments did convey appreciation towards the 4D UGET approach. These comments included improved comfort/satisfaction due to reduced duration of speculum insertion, being able to have an empty bladder and the reassurance of being able to see the transfer on the ultrasound screen.

No difference between the groups regarding the difficultly of ET was noted but all 4D UGET procedures were performed by the same experienced individual (LN). More conversions to 2D UGET were noted in the 4D UGET group, predominately due to suboptimal views in patients with an axial or thin endometrium. We are aware that inherent subjective bias could influence these results, therefore in a future study multiple practitioners should perform the two ET techniques that are compared. Although the 4D UGET did take a significantly longer time to perform and this could be at the detriment to a busy ET list, we feel the positive responses with regards to the 4D UGET would serve as justification to the small increase in length of procedure.

### Strengths and limitations

We conducted a large RCT with sufficient power to identify a significant difference between CPR with 4D UGET vs CTT, including an unselected population of 320 women recruited and randomised on the day of their ET.

A statistical difference was noted between the ages of the women in the per protocol analysis, but the marginal difference in mean ages (33 and 34 years), is unlikely to be clinically relevant. The ITT analysis revealed no significant difference in ages.

A curved catheter was required to perform 4D UGET, as the curve in the catheter helps to stabilise the catheter upon removal of the speculum and insertion of the TV ultrasound probe. Without the curve and bung the speculum could not be removed allowing the TV ultrasound probe to be re-introduced. Thus, the catheters utilised in our control and study groups were different. The differing catheters may pose an inherent bias in our data; however, both of these catheters are soft catheters and previous studies have confirmed non-inferiority between different soft catheters^31–35^.

A 1–2 s time lag between the 4D ultrasound images was noted during the trial and had to be accounted for when performing the transfer. We overcame this issue with slow and steady catheter insertion, and good communication between the performing practitioner and the embryologist. Future advances in ultrasound technology are expected to further refine this minor issue.

4D ultrasonography is an advanced technique and is not a skill available in every assisted reproductive technology (ART) unit. Additional training is required to develop the technique for performing this type of ultrasonography and it is currently untested against 2D UGET. Therefore, considering the additional cost and resource implications, we are cautious in recommending its routine use.

All of the 4D UGET and 95% of the CTT were performed by one experienced operator and although this reduced operator bias, we are not able to determine the effect or experience of different practitioners on the outcomes. We documented the duration of the procedure from the start until deposition of the embryo, however, the length of time from when the embryo leaves culture until it is deposited in the uterine cavity may be of more relevance as a surrogate of stress to the embryo.

## Conclusion

Our study has shown that 4D UGET significantly improves CPR, LBR and BPR compared with CTT. Future studies are warranted to assess the potential advantage of 4D UGET over 2D UGET to ensure the best ET methodology is utilised to achieve the highest LBR. Implementation of 4D UGET to routine practice would require the development of training programs suitable for upskilling ART practitioners with the view to improving ET outcomes as well as enhancing diagnosis of other relevant pathologies in reproductive medicine. Enactment of these advanced skills into routine care could be incorporated into a national guideline to ensure provision of best possible care for the patient.

## Methods

### Study design

This was a prospective randomised controlled (unblinded) parallel trial (RCT) comparing two techniques for ET (4D UGET vs CTT) conducted in a single, National Health Service (NHS) fertility centre in the UK between July 2018 and December 2019. The Hewitt Fertility centre is one of the largest reproductive medicine units in the UK, performing around 2,000 IVF cycles per annum.

### Sample size

In 2015, our unit trialled the 4D UGET technique on an unselected population of 50 patients. The CPR for this group was 40% versus the standard unit CPR of 25% (CTT ET performed).

The sample size was calculated based on data from this feasibility trial. Assuming an expected CPR in the intervention group of 40% and the control group of 25%, to achieve an 80% power to detect this expected difference, (with a significance level of 5%), 149 subjects per group would be required. With an estimated withdraw/non-evaluable subject rate of 5%, we aimed to recruit a total of 157 subjects per group, leading to a total required sample size of 314 subjects (final recruitment n = 320). Recruitment commenced in July 2018 and finished in December 2019, patients were followed up until they achieved a live birth, if they conceived from the ET.

### Study population

Consecutive unselected women were assessed for eligibility on the day of ET and approached for their participation if inclusion criteria were met (Table 4).

**Table 4:** Inclusion and exclusion criteria.

Inclusion criteria	Exclusion criteria
Undergoing fresh or frozen single blastocyst ET	Known or suspected hydrosalpinx
Able to provide written informed consent	Fluid within the endometrial cavity
	Gross distortion of endometrium (e.g. fibroids, bicornuate uteri)
	Previous myomectomy
	Previous randomisation
	Significant health issues, e.g. HIV, Hepatitis C, Hepatitis B, previous trachelectomy
	> 1 embryo transferred

Exclusion criteria were agreed upon to limit the number of variables that could affect pregnancy outcomes, ensuring the most homogenous data set. The presence of hydrosalpinx, or distortion of the endometrial cavity are known to negatively impact implantation rates^36–40^, previous myomectomies increase the risk of intrauterine adhesions, known to inhibit embryo implantation^41^; those with significant health issues are more likely to have failed implantation or early pregnancy loss^42^; and transferring more than one embryo is known to increase pregnancy rates but also multiple pregnancy rates and their associated complications^43^. Women were only randomised and enrolled to the RCT on one occasion. No embryos that were transferred had undergone preimplantation genetic testing (PGT).

### Randomisation

The patients were randomised after informed consent was taken on the day of ET. Patients were randomised to the study or control group using computer generated numbers created from an online randomisation tool (https://www.sealedenvelope.com/simple-randomiser/v1/lists)^44^ and were centrally distributed in a consecutive manner in sealed opaque envelopes prepared by the research team. The clinician and patient were made aware of the transfer method prior to entering the procedure room since equipment set up is different (one requires an ultrasound machine the other does not) and the patient preparation is different (empty versus full bladder).

### Control group

Women in the control group underwent ET according to the accepted standard practice in the unit during the study period, the CTT. The soft, Wallace Classic ET Catheter (18 or 23 cm) with centimetre graduations (Smiths Medical International Ltd, UK, CE marked) was utilised as first line. Stylets were used according to clinical requirements.

### Intervention group

**Figure 4:**
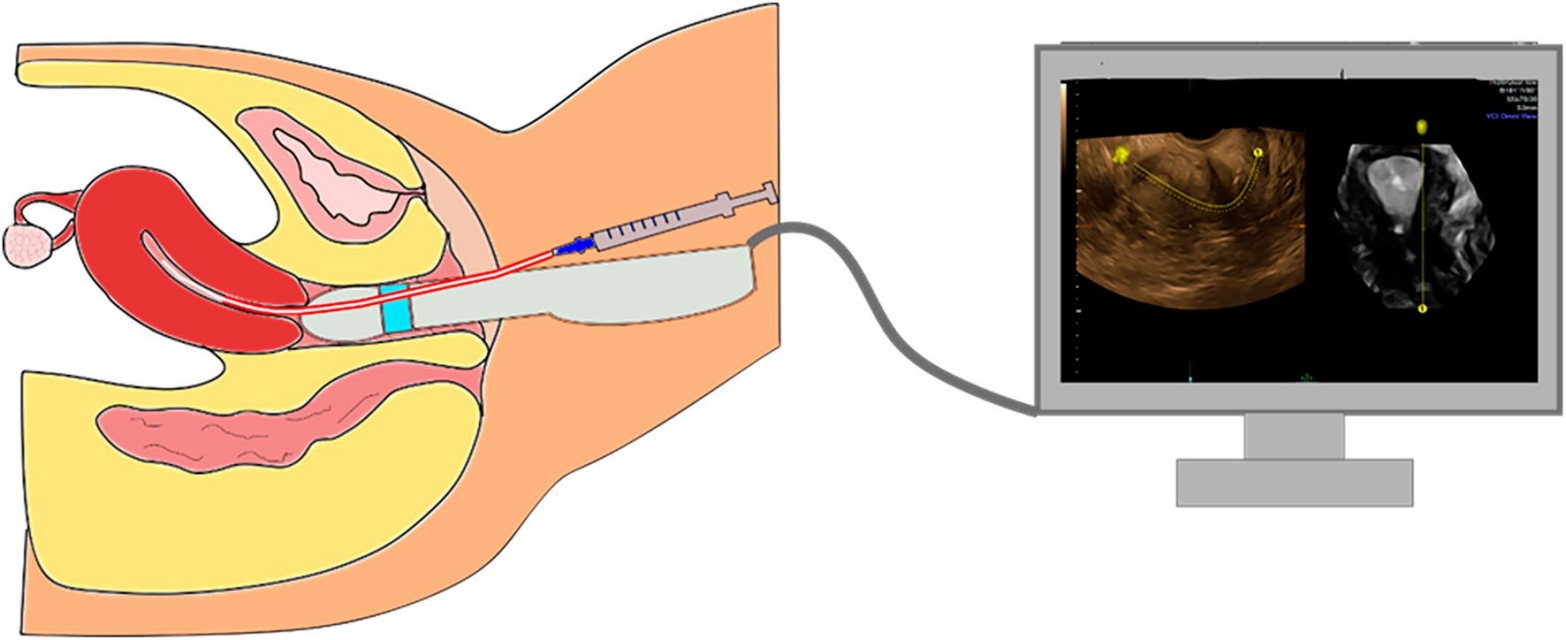
Figure depicting the ET process using the Kitazato catheter and 4D UGET.

The Kitazato ET Catheter Inner 3Fr. 40 cm Guide 30°/20 cm. Ref 223340 (CE 0086 International (Single Use) Kitazato Medical Co. Tokyo) has been designed to allow replacement of the embryo under transvaginal ultrasound guidance. Ultrasound scans were performed using a General Electric Volusen E8 ultrasound machine with a 3D/4D RIC5-9-D transvaginal probe (GE Medical Systems Kretztechnik GmbH & Co, Austria) (Fig. 4). The women in the intervention group were asked to empty their bladder prior to the procedure (to optimise the view of a transvaginal scan). Optilube (Optimum Medical solutions Ltd, UK) was used under a latex (Pasante, Pasante Healthcare Ltd, UK) or latex free (Non-latex Pasante, Pasante Healthcare Ltd, UK) probe cover (dependent on allergies).

### Ethical approval

This RCT was approved by the Liverpool central research ethics committee (REC ref: 16/NW/0588, IRAS 202857) and was registered to International Standard Randomised Controlled Trial Number registry (ISRCTN79955797, 06/02/2018, https://www.isrctn.com/ISRCTN79955797). All methods were performed in accordance with the relevant guidelines and regulations.

### Outcomes

#### Clinical

The primary outcome measure was CPR (presence of intrauterine pregnancy with fetal heart rate > 100 bpm between 6 and 8 weeks pregnancy). The secondary outcomes included LBR, biochemical pregnancy rate (BPR—defined as urinary pregnancy test positive), miscarriage, pregnancy of unknown location (PUL) and ectopic pregnancy rates. Duration of the procedure was recorded from start of patient preparation until embryo deposition.

#### Patient satisfaction

All participants completed a questionnaire after their ET. The women graded comfort and satisfaction associated with the procedure on a numerical scale from 1 to 5 (1 corresponded to being extremely uncomfortable or unsatisfied and 5 to being very comfortable and satisfied). The questionnaire also contained a free text comments section for participating respondents to document their judgments and views on the procedure.

#### Clinician satisfaction

Clinicians ranked the ease of the above procedures from 1 to 5, with 1 corresponding to being uncomplicated and straightforward and 5 being very difficult.

### Statistical analysis

All data was entered and analysed in the Statistical package for the Social Sciences (SPSS) for Windows (Version 26; IBM Corporation, USA). A per protocol and intention to treat (ITT) analysis was completed. Continuous data was analysed using the student`s *T*-test whilst categorical data was analysed using the χ^2^ test. Significance was achieved when the two-sided p-value was less than 0.05. The effect of the confounding factors of age and BMI on the results were calculated using logistic regression.

### Supplementary Information

Supplementary Figure 1.

## Data Availability

The dataset generated and/or analysed during this current study are not publicly available as the raw dataset has incorporated with participants identifiable information. Data are however available from the corresponding author on reasonable request.
