# Supplementary material for: Four-dimensional ultrasound guided embryo transfers improve live birth rates when compared to the clinical touch technique: a randomised controlled trial
Source: Sci Rep. 2023 Sep 8;13:14875. doi: 10.1038/s41598-023-41313-z (PMC10491625; doi:10.1038/s41598-023-41313-z)
Supplement: Supplementary file 1 — Supplementary Figure 1. [file 41598_2023_41313_MOESM1_ESM.pdf]

Supplementary figure 1. Day 5 embryo grading matrices to assign embryos into good, average and poor-quality categories.

| Key |
|---|
| Good |
| Average |
| Poor |

## Day 5 Embryo Grading Scheme

| | | Inner cell mass/Trophectoderm grade | | | | | | | | | | | |
|---|---|---|---|---|---|---|---|---|---|---|---|---|---|
| | | Aa | Ab | Ac | Ba | Bb | Bc | Ca | Cb | Cc | Da | Db | Dc |
| Expansion status | 1 | 1Aa | 1Ab | 1Ac | 1Ba | 1Bb | 1Bc | 1Ca | 1Cb | 1Cc | 1Da | 1Db | 1Dc |
| | 2 | 2Aa | 2Ab | 2Ac | 2Ba | 2Bb | 2Bc | 2Ca | 2Cb | 2Cc | 2Da | 2Db | 2Dc |
| | 3 | 3Aa | 3Ab | 3Ac | 3Ba | 3Bb | 3Bc | 3Ca | 3Cb | 3Cc | 3Da | 3Db | 3Dc |
| | 4 | 4Aa | 4Ab | 4Ac | 4Ba | 4Bb | 4Bc | 4Ca | 4Cb | 4Cc | 4Da | 4Db | 4Dc |
| | 5 | 5Aa | 5Ab | 5Ac | 5Ba | 5Bb | 5Bc | 5Ca | 5Cb | 5Cc | 5Da | 5Db | 5Dc |
| | 6 | 6Aa | 6Ab | 6Ac | 6Ba | 6Bb | 6Bc | 6Ca | 6Cb | 6Cc | 6Da | 6Db | 6Dc |
